# Comparing the Harmonic Scalpel with Electrocautery in Reducing Postoperative Flap Necrosis and Seroma Formation after Modified Radical Mastectomy in Carcinoma Breast Patients: A Double-Blind Prospective Randomized Control Trail

**DOI:** 10.7759/cureus.2476

**Published:** 2018-04-13

**Authors:** Arumugom Archana, Sathasivam Sureshkumar, Chellappa Vijayakumar, Chinnakali Palanivel

**Affiliations:** 1 Surgery, Jawaharlal Institute of Postgraduate Medical Education and Research (JIPMER), Puducherry, India.; 2 Preventive Medicine, Jawaharlal Institute of Postgraduate Medical Education and Research (JIPMER), Puducherry, India.

**Keywords:** breast carcinoma, electrocautery, flap necrosis, seroma, mastectomy, harmonic scalpel, surgical site infections, quality of life

## Abstract

Introduction

Only a few studies compare the efficacy of the harmonic scalpel and electrocautery in performing mastectomies, and these have mainly compared their intraoperative parameters. But the main concern with electrocautery is the incidence of flap necrosis and seroma formation. Therefore, this study was done to determine if the harmonic scalpel has any advantages over electrocautery in reducing postoperative flap necrosis and seroma formation in patients undergoing a modified radical mastectomy (MRM).

Methodology

This randomized control trial was carried out over a one-year period in a tertiary care centre in South India. The study patients were randomized into an electrocautery group and a harmonic scalpel group. In the first group, mastectomy including flap and axillary dissection was done using electrocautery. In the second group, a harmonic scalpel was used for dissection. This study compared the efficacy of the harmonic scalpel with electrocautery in terms of postoperative seroma formation and flap necrosis. Various other perioperative parameters like the number of drain days, total drainage volume (in mL), operating time (in minutes), intraoperative blood loss (in mL), and postoperative wound site pain were also studied. During each postoperative visit, the presence of seroma was assessed clinically, and the number of aspirations required for the seroma was also analysed.

Results

A total of 240 patients were randomized into two groups of 120 patients each. Baseline parameters were comparable across both groups. There were significant differences in the duration of surgery [151.38 mins vs. 112.33 mins; p = 0.001] and intraoperative blood loss [276.25 mL vs.200.13 mL; p = 0.001]. On Postoperative Day (POD) 1, the difference in the mean pain scores [6 vs. 4; p = 0.001] was statistically significant. In addition, the differences in the mean total drainage volume [937.5 mL vs. 470 mL; p = 0.002] and the incidence of seroma during the first follow-up [34.2% vs. 21.7 %; p = 0.030] were statistically significant. The difference in the incidence of flap necrosis on POD 4 [7.5% vs. 4.2%; p = 0.2706] was not statistically significant. None of the patients developed flap necrosis after four days.

Conclusion

The harmonic scalpel reduces the total drainage volume of seromas, the number of drain days, intraoperative blood loss, duration of surgery, postoperative pain (especially on POD 1 and 5), and incidence of flap necrosis.

## Introduction

Scalpels with disposable knives are traditionally used for mastectomy procedures. In the last two decades, electrocautery has been accepted as a safe alternative to the scalpel in terms of reduced blood loss and less operating time [1]. However, the increased incidence of seroma formation and flap necrosis is still a concern for many surgeons who prefer to use cold knives [2]. 

The introduction of the ultrasonically activated harmonic scalpel a decade ago was a major breakthrough in the field of surgery; it is now considered an integral part of all advanced laparoscopic procedures [3]. Though the harmonic scalpel is used in many other major open operations, its use in modified radical mastectomy (MRM) is still limited.

An electrocautery performs its action via an electrical current that produces heat. The harmonic scalpel uses ultrasound technology to create high-frequency mechanical vibrations in the range of 20,000Hz to 60,000Hz to cut and coagulate at the same time, sealing vessels at lower temperatures than electro-surgery. The vibrations cut through tissue and seal it using protein denaturisation, rather than heat [4].

The harmonic scalpel has the advantages of precise dissection, reliable hemostasis, less lateral thermal spread, and less charring, surgical smoke, desiccation, and tissue sticking when compared with electrocautery [5]. The harmonic scalpel is not without constraints, which include cost and availability limitations [6].

There are only a few studies comparing the harmonic scalpel and electrocautery in MRM, and they mainly compare intraoperative parameters, though the primary concern is the incidence of flap necrosis and seroma formation with electrocautery [7]. Therefore, this study was done to determine if the harmonic scalpel offers any advantages in reducing postoperative flap necrosis and seroma formation in patients undergoing MRM when compared with electrocautery.

## Materials and methods

This double-blind prospective randomized controlled trial was carried out over the period of one year in a tertiary care centre in South India. This trial included all patients with an operable carcinoma breast planned for MRM. The study excluded patients with ulcers, discharge, or active wound infections in the breast or anywhere in the body. The study also excluded patients with risk factors that could affect wound healing like anemia or on anticoagulant or corticosteroid therapy. Institute Human Ethics Committee (IEC) approval was obtained for the study. The nature, methodology, and risks involved in the study were explained to the patient and informed consent was obtained. All the information collected was kept confidential, and the patient was given full freedom to withdraw at any point during the study. All provisions of the Declaration of Helsinki were followed in this study.

Patient characteristics including age, body mass index (BMI), clinical staging of the cancer, and details of the neo-adjuvant therapy received and co-morbidities were recorded. Primary outcome parameters like the incidence of postoperative seroma formation and flap necrosis were noted. The number of drain days (number of days drain kept per patients) and total drainage volume (in mL) were also noted. Secondary outcome parameters were operating time (in minutes), intraoperative blood loss (in mL), and postoperative wound site pain. Patients were randomized into two groups (electrocautery group and harmonic group).

On the day of surgery, patients were operated using either electrocautery or the harmonic scalpel. In the electrocautery group, the mastectomy including flap and axillary dissection was done using an electro-surgical unit (ESU), ErbeVio 300 D (Erbe Medical India, Chennai, India), set at both cutting and coagulation mode as desired, delivering a 350 kHz sinusoidal current. In the harmonic scalpel group, the synergy blade along with harmonic shear (Generator 300, Ethicon Endosurgery Inc., Cincinnati, USA) was used. The principal investigator collected the intraoperative parameters with the help of a surgery resident who assisted the surgery, and the postoperative parameters with the help of another competent consultant surgeon.

Operating time in minutes from skin incision to closure was recorded. Blood loss during the surgery was estimated by weighing the dry sponges preoperatively and subtracting the weight from the weight of postoperative used sponges (using a digital weighing scale with each gram taken as equal to one millilitre of blood). Suction was not used in the surgery. The amount of blood loss was calculated as mL. Two drains were placed, one under the flap and the second in the axilla.

Postoperatively, wound-related pain was assessed using visual analogue scales (VAS) scored on each postoperative morning till Postoperative Day (POD) 5. Clinical assessment of the wound was done on each postoperative morning for flap necrosis and surgical site infection (SSI) till the patient was discharged and on the first follow-up visit to the hospital. The volume of suction drain (in mL) on each POD till the drain was removed and the duration of drain was documented. The drains were removed if the volume was less than 30 mL in 24 hours. Patients discharged after the removal of drains were followed up weekly for up to four weeks. During each postoperative visit, the presence of seroma was assessed clinically, and the number of aspirations required for the seroma was also documented. 

Statistical analysis

Based on the assumed SSI rate of 30% in class IV dirty abdominal operative wounds by primary closure and an expected reduction in the infection rate by 15% with delayed primary closure, considering the alpha error of 5%, power of 80%, and expected drop out rate of 10%, the sample size was calculated to be 120 in each group. P value <0.05 was considered as significant. All categorical data between both groups were compared using the Chi-square test or Fischer’s exact test. The data related to continuous variables were compared using independent student t-test.

## Results

A total of 240 patients were included in the study, of whom 120 patients (50%) underwent MRM by electrocautery and 120 patients (50%) by harmonic scalpel. Both groups were comparable with respect to age and the presence of co-morbidities. The stages of breast cancer at which patients underwent MRM were comparable between the study groups (Table 1).

**Table 1:** Baseline demographic parameters in the study groups SD: Standard deviation; N: Number; DM: Diabetes; HTN: Hypertension

Demographic parameters		Electrocautery (n=120)	Harmonic scalpel (n=120)
Mean age (Mean + SD)		58.72 + 10.69	58.70 + 9.85
Co-morbidities [N (%)]	Absent	70 (58.3%)	72 (60%)
	DM	19 (15.8%)	18 (15%)
	HTN	25 (20.8%)	23 (19.2%)
	DM+HTN	5 (4.2%)	5 (4.2%)
	Thyroid	1 (0.8%)	2 (1.7%)
Breast cancer	Stage l	12 (10%)	13 (15.6%)
	Stage ll	72 (60%)	68 (56.7%)
	Stage lll	36 (30%)	38 (31.7)

In this study, the difference in duration of surgery [151.38 mins vs. 115.84 mins; p = 0.001] and intraoperative blood loss [276.25 mL vs.200.13 mL; p = 0.002] between the two groups was statistically significant (Table 2).

**Table 2:** Comparison of intraoperative parameters between the study groups SD: Standard deviation

Intra-operative parameters (Mean + S.D)	Electrocautery (n = 120)	Harmonic scalpel (n = 120)	p-value
Duration of surgery (mins)	151.38 + 31.89	112.33 + 19.35	0.001
Blood loss (mL)	276.25 + 108.10	200.13 + 65.19	0.002

**Figure 1:**
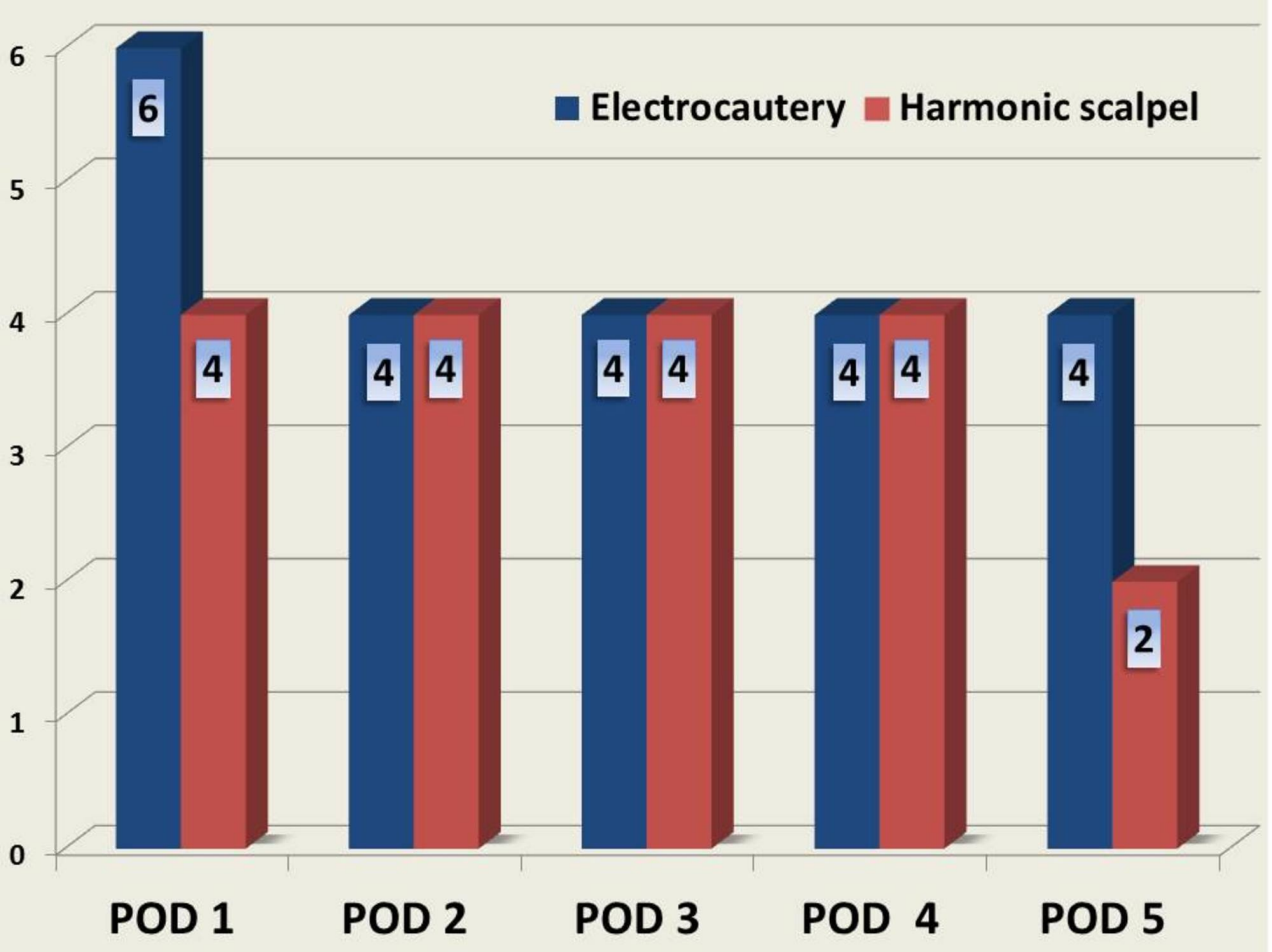
Comparison of postoperative pain scores between the study groups POD: Postoperative day

On POD 1, the difference between the mean VAS pain scores of the two groups [6 vs. 4; p = 0.001] was statistically significant. But on POD 2, POD 3 and POD 4, this difference [4 vs. 4; p = 0.0538] was statistically not significant. On POD 5, the difference in mean VAS pain scores between the groups [4 vs. 2; p = 0.002] was statistically significant (Figure 1).

The difference in the mean number of drain days [8 vs. 6.5; p = 0.001], mean total drainage volume  [937.5 mL vs. 470 mL; p = 0.002], and incidence of seroma during the first follow-up among the groups [34.2% vs. 21.7%; p = 0.030] were statistically significant. The difference in the incidence of seroma during the second follow-up between the groups [6.7% vs. 5%; p = 0.784] was not statistically significant. None of the patients had seroma in the third and fourth follow-up visits (Table 3).

**Table 3:** Comparison of postoperative parameters between the study groups N: Number

Postoperative parameters	Electrocautery (n = 120)	Harmonic scalpel (n = 120)	p-value
Mean drain days (N)	8	6.5	0.001
Mean drain volume (mL)	937.5	470	0.002
Incidence of seroma [N (%)]	41 (34.2%)	26 (21.7%)	0.030
Incidence of edge necrosis [N (%)]	12 (10%)	5 (4.2%)	0.0782

**Figure 2:**
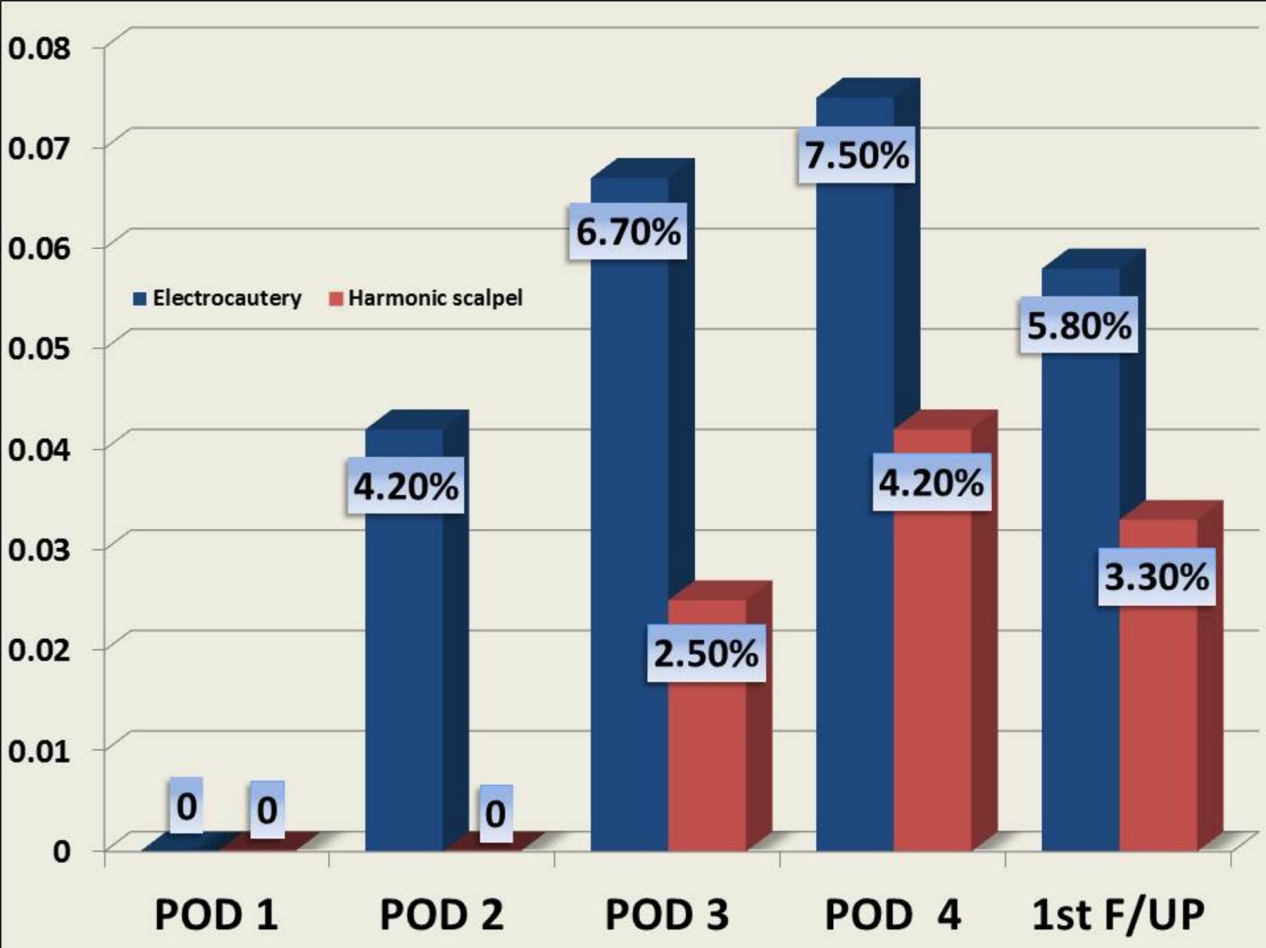
Incidence of flap necrosis in the study groups POD: Postoperative day; F/UP: Follow-up

The difference in the incidence of flap necrosis (which is inclusive of edge necrosis) [10% vs. 4.2%; p = 0.0782] was statistically not significant. None of the patients developed flap necrosis on POD 1. The difference in the incidence of flap necrosis on POD 2 [4.2% vs. 0%; p = 0.0499] was statistically significant. The difference in the incidence of flap necrosis on POD 3 [6.7% vs. 2.5%; p = 0.12] and on POD 4 [7.5% vs. 4.2%; p = 0.2706] was not statistically significant. None of the patients developed flap necrosis after four days (Figure 2).

The difference in the incidence of flap necrosis during the first follow-up visit [5.8% vs. 3.3%; p = 0.53] was not statistically significant. The difference in the incidence of flap necrosis during the second follow-up [4.2% vs.0%; p = 0.0499] was statistically significant. None of the patients in both groups had flap necrosis in their third and fourth follow-up visits.

## Discussion

The harmonic scalpel has become an integral part of various surgeries owing to its advantages like precise dissection, reliable haemostasis, and relatively lesser tissue damage. However, its use in MRM is not yet well established [8]. There is a dearth of studies in the literature comparing the use of the harmonic scalpel and electrocautery in MRM. Studies have reported the advantages of the harmonic scalpel compared to electrocautery in terms of various intraoperative parameters; however, reports on the incidence of postoperative seroma formation and flap necrosis following harmonic scalpel and electrocautery MRM [9]. Therefore, a randomized controlled trial was planned to study the same.

Parveen S et al. and Sarwar G et al. have demonstrated significantly lower intraoperative blood loss with the harmonic scalpel when compared to electrocautery in patients undergoing MRM [10-11]. Similar results were obtained in the current study. The lower blood loss associated with the harmonic scalpel can be attributed to its principle of using high-frequency vibrations to seal vessels at a lower temperature compared to electrocautery.

The studies by Parveen S et al. and Galatius H et al. reported a significant decrease in intraoperative time with the harmonic scalpel when compared to electrocautery [10, 12]. However, a few studies have shown no significant difference in operating time [12-13]. The present study has also demonstrated that there is a significant difference in the duration of surgery when the harmonic scalpel is used, versus electrocautery. This study also found that postoperative wound site pain on POD 1 and 5 was significantly lesser in case of use of harmonic scalpel when compared to electrocautery, which is possibly due to lesser tissue damage.

The studies by Parveen S et al. and Porter KA et al. show that the number of drain days and total drainage volume was lesser with the use of the harmonic scalpel when compared to electrocautery [10, 14]. The current study also reported similar results with respect to drain days and volume. The harmonic scalpel uses ultrasound waves to seal lymphatic vessels which do not open again, unlike in electrocautery; this significantly reduces the drain volume and the number of drain days.

The effect of the harmonic scalpel in reducing flap necrosis and seroma formation post MRM is not widely studied. Rebeiro et al. reported a lower incidence of flap necrosis with the use of the harmonic scalpel compared to electrocautery [15]. This study has shown a statistically significant decrease in the incidence of flap necrosis between the two groups. The reason for the decreased incidence of seroma in both groups might be the adherence of the flap to the skin and the sealing of lymphatic vessels. Rodd CD et al. and Rebeiro GH et al. in their studies demonstrated no significant difference in seroma formation between the two groups [13,15]. The current study also had similar results.

Limitations

Harmonic scalpel surgery involves the use of hand probes which increases the cost of the surgical procedure compared to electrocautery. Hence, it is prudent to assess the total cost involved in the treatment of these patients with respect to the reduction in operating time and length of hospitalization, etc. However, being a public sector institution, this study could not evaluate the same as healthcare is offered to patients here free of charge. Further studies focusing on the cost-benefit balance may help in addressing this issue.

## Conclusions

On comparing the harmonic scalpel with electrocautery in reducing postoperative flap necrosis and seroma formation after MRM, it was found that the harmonic scalpel reduces the total drainage volume of seromas, the number of drain days, intraoperative blood loss, the duration of surgery, postoperative pain, and the incidence of flap necrosis.
